# Post-hepatectomy venous thromboembolism: a systematic review with meta-analysis exploring the role of pharmacological thromboprophylaxis

**DOI:** 10.1007/s00423-022-02610-9

**Published:** 2022-07-26

**Authors:** Monish Karunakaran, Ramneek Kaur, Simi Ismail, Sushma Cherukuru, Pavan Kumar Jonnada, Baiju Senadhipan, Savio George Barreto

**Affiliations:** 1 Department of Surgical Gastroenterology, Asian Institute of Gastroenterology, Hyderabad, India; 2 College of Medicine and Public Health, Flinders University, Adelaide, South Australia Australia; 3 Department of Anesthesiology, Institute of Liver Transplantation & Regenerative Medicine, Medanta-The Medicity, Gurugram, India; 4 Department of Radiology, KIMS Hospital, Thiruvananthapuram, India; 5 Department of Pathology, ESI Hospital, Hyderabad, India; 6 Department of Surgical Oncology, Basavatarakam Indo-American Cancer Hospital & Research Institute, Hyderabad, India; 7 Division of Surgery and Perioperative Medicine, Flinders Medical Center, Bedford Park, Adelaide, South Australia Australia

**Keywords:** Hepatectomy, Outcomes, Venous thromboembolism, Hemorrhage, Quality

## Abstract

**Purpose:**

Patients undergoing hepatectomy are at moderate-to-high risk of venous thromboembolism (VTE). This study critically examines the efficacy of combining pharmacological (PTP) and mechanical thromboprophylaxis (MTP) versus only MTP in reducing VTE events against the risk of hemorrhagic complications.

**Methods:**

A systematic review of major reference databases was undertaken, and a meta-analysis was performed using common-effects model. Risk of bias assessment was performed using Newcastle–Ottawa scale. Trial sequential analysis (TSA) was used to assess the precision and conclusiveness of the results.

**Results:**

8 studies (*n* = 4238 patients) meeting inclusion criteria were included in the analysis. Use of PTP + MTP was found to be associated with significantly lower VTE rates compared to only MTP (2.5% vs 5.3%; pooled RR 0.50, *p* = 0.03, *I*^2^ = 46%) with minimal type I error. PTP + MTP was not associated with an increased risk of hemorrhagic complications (3.04% vs 1.9%; pooled RR 1.54, *p* = 0.11, *I*^2^ = 0%) and had no significant impact on post-operative length of stay (12.1 vs 10.8 days; pooled MD − 0.66, *p* = 0.98, *I*^2^ = 0%) and mortality (2.9% vs 3.7%; pooled RR 0.73, *p* = 0.33, *I*^2^ = 0%).

**Conclusion:**

Despite differences in the baseline patient characteristics, extent of hepatectomy, PTP regimens, and heterogeneity in the pooled analysis, the current study supports the use of PTP in post-hepatectomy patients (grade of recommendation: strong) as the combination of PTP + MTP is associated with a significantly lower incidence of VTE (level of evidence, moderate), without an increased risk of post-hepatectomy hemorrhage (level of evidence, low).

**Supplementary Information:**

The online version contains supplementary material available at 10.1007/s00423-022-02610-9.

## Introduction 

VTE acquired during hospitalization is a significant contributor to morbidity, and even mortality, in patients undergoing major abdominal surgery. In a study that included 183,069 patients in general surgery, an episode of VTE was found to increase the 30-day mortality by nearly 9% (11.19% vs 2.54%; *p* = 0.0001) [1]. Furthermore, two recent reviews noted that the average cost of a VTE event ranged between $12,000 and $15,000 per patient, leading to an annual estimated healthcare system cost of $7–10 billion in USA [2, 3]. The Agency for Healthcare Research and Quality has advised that VTE prophylaxis is amongst the top 10 suggested practices for improving patient safety [4]. Appreciating its importance, PTP is recommended for patients undergoing general surgical procedures with at least moderate risk (≥ 3%) of VTE [5].

While the efficacy and safety of LMWH in preventing VTE in general surgery patients are established [6], there is a lack of convincing evidence for the same in patients undergoing hepatectomy. VTE events post-hepatectomy are not uncommon with an overall incidence of DVT between 2.1 and 4.3% with attendant PE incidence rates of 1.4–7.1% [7–10]. De Martino et al. [11] noted that patients undergoing hepatectomy had the highest risk of VTE amongst all abdominal surgical procedures. Post-hepatectomy VTE is associated with major morbidity, including acute renal failure, pneumonia, sepsis, re-intubation, prolonged ventilation, and cardiac arrest, significantly increased length of post-operative stay, and even mortality [12]. These findings prompted Newhook et al. [12] to stress the importance of routine PTP to obviate the risk of VTE. Most patients undergoing hepatectomy are considered moderate-to-high risk for VTE (3.0%) [13] based on the modified Caprini risk scoring system [14]. However, on account of the low quality of evidence, the American College of Chest Physicians in 2012 issued a grade 2C recommendation for peri-operative PTP, depending on the patient’s perceived risk for bleeding [5].

A previously published systematic review and meta-analysis on the topic has demonstrated a benefit of PTP in reducing the incidence of VTE events following hepatic resection [15]. However, the study did not address the perceived increased risk of bleeding as a result of PTP. This systematic review aims to interrogate the available evidence to confirm the benefit of PTP in addition to mechanical thromboprophylaxis (MTP) versus only MTP in the prevention of post-hepatectomy VTE as well as determine its safety with respect to bleeding complications and also its impact on length of stay and mortality.

## Study methodology

### Search strategy

**Fig. 1:**
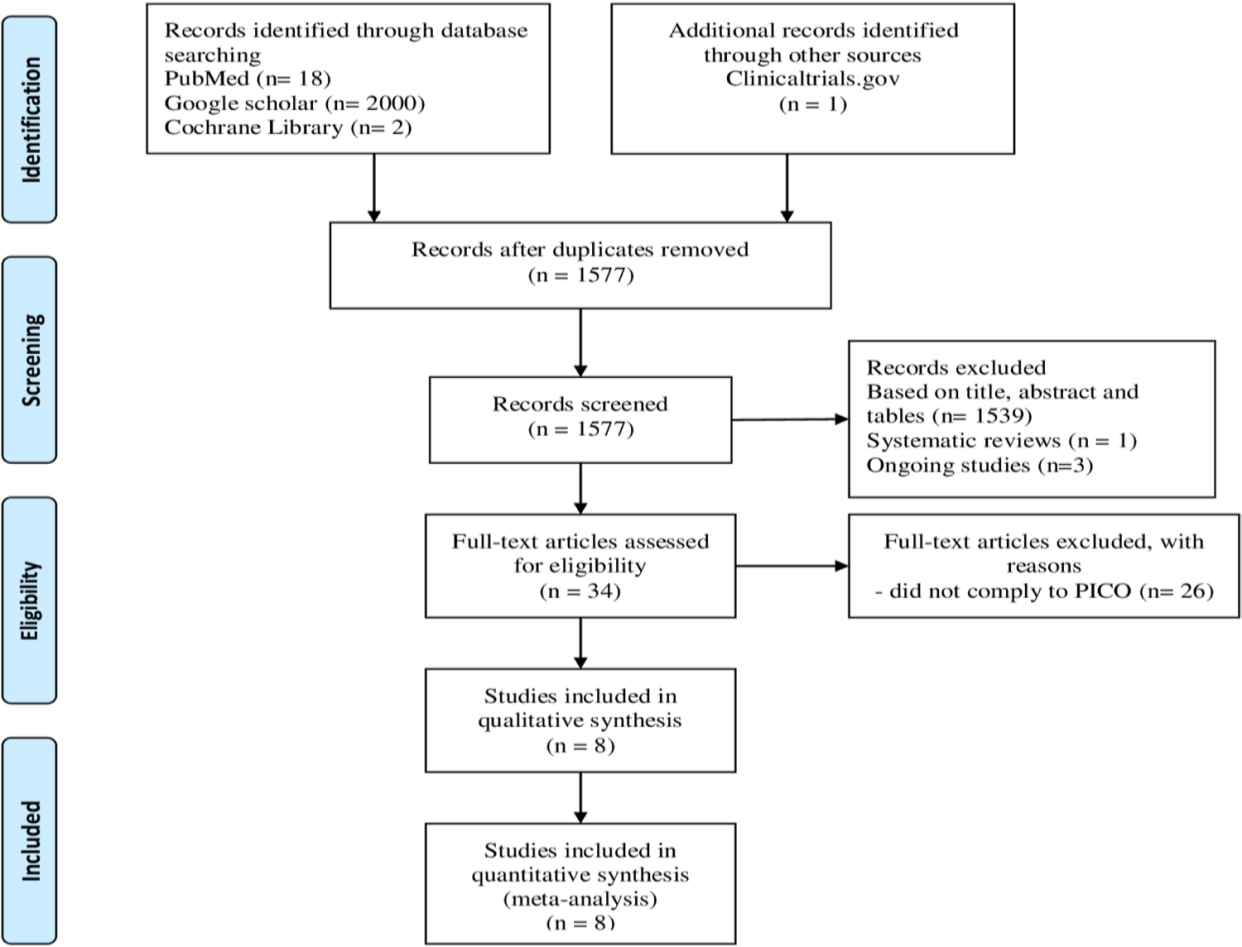
PRISMA flow diagram for selection of studies

We performed a systematic literature search using Medline, Embase, PubMed, Scopus, and Google Scholar for full text articles published between 2000 and November 2021 using the following MeSH keywords (Supplementary Table 1): "Venous thromboembolism" OR "Deep vein thrombosis" OR "Pulmonary Embolism" OR "Thromboembolic episode" AND "Thromboprophylaxis" OR "Pharmacological Thromboprophylaxis" OR "Chemothromboprophylaxis" AND "Hepatectomy" OR "Hepatic Resection" OR "Liver Resection." The references of included articles were screened further to identify similar additional studies. All aspects of the PRISMA guidelines [16] were strictly adhered to while searching and reporting the articles (Fig. 1).

The authors (MK and RK) independently employed a three-step search strategy. An initial limited search was undertaken, followed by an analysis of the text words contained in the title and abstract and of the index terms used to describe articles. A second search using all identified keywords and index terms was then undertaken across all included databases. Thirdly, the reference lists of articles and “related articles” function was perused for similar additional articles. All the screened articles were assessed for eligibility, and any disagreement was resolved through mutual discussion. The accuracy of the extracted data was adjudicated further by the senior author (SGB). The study has been registered with PROSPERO (CRD42022288658).

### Inclusion criteria

Studies fulfilling the following PICOS criteria were deemed eligible for inclusion in the systematic review:

- P (Population): Patients undergoing hepatectomy
- I (Intervention of interest): Peri-operative PTP, in addition to mechanical TP (PTP + MTP)
- C (Comparator): only MTP
- O (Outcomes): The review will identify and synthesize study results on the following in, both, the intervention and comparator arms:
  - Primary: Post-hepatectomy VTE (includes DVT, PE, and portal vein thrombosis (PVT))
  - Secondary: (a) Post-hepatectomy hemorrhage; (b) Post-operative mortality; (c) Post-operative length of hospitalization
- S (Study design): Randomized controlled trials as well as non-randomized observational, comparative studies

All the included studies were conducted at high-volume liver surgery centers, defined as centers performing > 20 liver resections annually [17].

### Exclusion criteria

- Studies that did not have a comparative analysis (PTP + MTP versus MTP)
- Outcomes of interest (post-hepatectomy VTE, hemorrhage, mortality, or length of hospitalization) were not reported
- Inability to extract relevant data from the published results

### Data extraction and quality assessment

For included studies, two authors (MK and RK) extracted the data using the agreed form. For each study that fulfilled the criteria, the following information was extracted: name of the first author, year of publication, study design, sample size, demographic variables, laboratory data including preoperative platelet count, INR and aPTT, indication for hepatectomy, presence and severity of cirrhosis, extent of hepatectomy (based on number of resected segments), strategy of PTP (drug, time of initiation and duration), operative duration, intra-operative blood transfusion, radiological protocols employed to diagnose VTE events and comparative outcomes including VTE rates, post-operative hemorrhage, length of hospitalization, morbidity, mortality, and re-admission rates.

Two authors (MK and RK) independently judged the quality of the included studies using the Newcastle–Ottawa scale [18]. Any discrepancies between authors were resolved by discussion and agreement. When required, the disagreements between the 2 authors were resolved by the senior author (SGB). The funnel plots were used to evaluate publication bias using linear regression test of funnel plot asymmetry.

### Statistical analysis

Statistical analysis was performed using R software, version 4.0.5 (R Foundation for Statistical Computing, Vienna, Austria). Continuous variables were analyzed by the Risk Ratio (RR), and 95% confidence interval (CI) was calculated using Mansel-Haenszel method. The heterogeneity of the included studies was assessed using *χ*^2^, *I*^2^ tests, and by Galbraith plot. *I*^2^ of 0–30, 30–60, 50–70, and > 75% represent low, moderate, substantial, and considerable heterogeneity, respectively. Studies with a *p*-value < 0.10 and *I*^2^ > 60% indicated substantial heterogeneity. If significant heterogeneity existed in the fixed-effects model, then analysis was done using random-effects model. The *p* value > 0.10 and *I*^2^ < 25% were considered for assessing statistical significance for heterogeneity. The *z*-test was used to determine the pooled RR, and the significance was set to reject the null hypothesis at *p* < 0.05. Leave-out analysis and influence analysis (Baujat plots) [19] were employed to explore significant heterogeneity. TSA was performed using SA software (0.9.5.5 Beta, Copenhagen Trial Unit, Copenhagen, Denmark) [20]. Random-effects models were used to compute pooled outcome data with 95% confidence level (2-sided CI) demonstrating statistical significance. The required information size (IS) was computed according to 10% relative risk reduction between the PTP and non-PTP groups and achievement of 80% power.

## Results

Table 1 summarizes the eight studies (three from the United States [9, 10, 21], two each from Japan [22, 23] and China [24, 25], and one from Italy [26]), including a total of 4238 patients, published between 2010 and 2021 that were suitable for inclusion. Details of the excluded studies [8, 12, 27–50] and the reasons for exclusion are provided in Supplementary Table 2. For study selection and data abstraction, there was complete (100%) agreement between the two authors (MK and RK), with a Cohen’s kappa statistic of 1. While one of the included studies [25] was prospective in design, the remaining 7 studies were retrospective in nature [9, 10, 21–24, 26]. Out of the 4238 patients included in the review, 2541 (60%) received PTP + MTP. The utilization of PTP + MTP varied widely between studies, from 18.9% in the study by Yamashita et al. [22] to 75% in the study by Ejaz et al. [21]. No study, except Ejaz et al. [21], administered pre-operative PTP (Table 2). All patients received routine MTP.

**Table 1:** Baseline characteristics of the individual studies included in the analysis (Abbreviations: aPTT—activated partial thromboplastin time; BMI—body mass index; INR—international normalized ratio; MTP—mechanical thromboprophylaxis; NOS—Newcastle–Ottawa Scale; P—prospective; PTP—pharmacological thromboprophylaxis; R—retrospective)

Study	Design	N	PTP + MTP versus only MTP							Hepatectomy (major/minor)	NOS
			n	Age	Gender (males, *n* (%))	BMI	Platelet count	INR	aPTT		
Vivarelli et al. (2010), Italy [26]	R	229	157 (68.5)/72 (31.5)	65.0 ± 9.8/63.0 ± 9.5 (*p* = 0.08)	119 (76.0)/52 (72.0) (*p* = 0.33)	-	150 ± 60/115 ± 51 (*p* = 0.001)	-	-	10/219	7
Reddy et al. (2011), USA [9]	R	419	275 (65.6)/144 (34.4)	58 ± 20/58 ± 21 (*p* = 0.36)	115 (41.8)/75 (52.1) (*p* = 0.06)	27.0 ± 6.1/26.4 ± 7.1 (*p* = 0.46)	233 ± 112/240 ± 114 (*p* = 0.85)	1.0 ± 0.1/1.0 ± 0.1 (*p* = 0.32)	30.0 ± 4.3/27.8 ± 6.0 (*p* < 0.001)	419/0	6
Nathan et al. (2014), USA [10]	P	2147	1295 (60.3)/852 (39.7)	60 (50–70)	1085 (51)	27 (24–31)	154 (119–193)	1.4 (1.2–1.6)	-	704/1443	5
Yamashita et al. (2014), Japan [22]	R	275	53 (18.9)/228 (81.1)	69 ± 10/65 ± 12 (*p* = 0.01)	32 (60)/169 (75) (*p* = 0.06)	24.4 ± 4/22.9 ± 3.1 (*p* < 0.01)	16.7 ± 5.1/16.4 ± 6.5 (*p* = 0.69)	-	-	53/224	7
Ejaz et al. (2014), USA [21	R	597	454 (76)/145 (24)	58 (50–68)/57 (47–64) (*p* = 0.05)	227 (50)/75 (51.7) (*p* = 0.72)	-	-	1 (0.9–1.0)/0.9 (0.9–1.0) (*p* < 0.001)	-	195/402	6
Hayashi et al. (2014), Japan*[23]	P	138	72 (52.2)/66 (47.8)	-	-	-	-	-	-	138	5
Wang et al. (2018), China [25]	P	233	117 (50.2)/116 (49.8)	58.52 ± 8.7/57.69 ± 8.4 (*p* > 0.05)	91 (77.8)/ 85 (73.3) (*p* > 0.05)	-	-	2.46 ± 0.44/2.65 ± 0.39 (*p* > 0.05)	30.26 ± 2.8/30.41 ± 3.5 (*p* > 0.05)	233	7
Ming-lei et al. (2021), China [24]	P	192	96 (50)/96 (50)	52.7 ± 12.9/ 50.9 ± 13.0 (*p* = 0.366)	65 (67.7)/66 (68.8) (*p* = 0.877)	23.3 ± 2.9/22.8 ± 3.3 (*p* = 0.328)	225 (180.5–265.5)/208 (155–275.5) (*p* = 0.315)	0.99 (0.96–1.08)/1.02 (0.96–1.08) (*p* = 0.306)	26.3 ± 3.9/25.9 ± 3.4 (*p* = 0.433)	192	7

^*^Hepatectomy cohort alone

**Table 2:** Thromboprophylaxis strategies in the included studies (Abbreviations: PTP—pharmacological thromboprophylaxis; MTP—mechanical thromboprophylaxis; UFH—unfractionated heparin; LMWH—low molecular weight heparin; POD—post-operative day; N/A—not Available)

Study	PTP strategy			Type of MTP
	Drug	Initiation time	Duration	
Vivarelli et al. [26]	Nadroparin calcium 0.3 ml/ Enoxaparin sodium 0.4 ml	From day of surgery	Until mobilization (≥ 7 days)	Anti-embolism stockings
Reddy et al. [9]	UFH (54.7%)/Enoxaparin (9.1%)/UFH + LMWH (sequential) (1.9%)	Median POD1	Not specified	Knee-high graduated compression stockings
Nathan et al. [10]	UFH LMWH	POD 0–1: 38% POD 2–5: 22% POD > 5: 40%	Median 5 days Median 6 days N/A	Intermittent pneumatic compression (IPC)
Yamashita et al. [22]	Enoxaparin	Within 24–36 h after liver resection, or 12 h after removal of epidural catheter	Until discharge (≤ 14 days)	Elastic compression stockings Intermittent pneumatic compression (IPC)
Ejaz et al. [21]	LMWH Enoxaparin	Pre-op: 68.2% Post-op (within 24 h): 90.2% Post-op (> 24 h): 9.8%	NA	Sequential compression devices (SCD)
Hayashi et al.*[23]	LMWH 4000 IU, twice daily Fondaparinux, 1.5 or 2.5 mg, once daily	After 24 h of surgery	Till POD 8	Elastic stockings (ES) and intermittent pneumatic compression (IPC)
Wang et al. [25]	LMWH 5000 U, once daily	POD 2	POD 7	Lower limb activity on the bed and to do out-of-bed activity as early as possible
Ming-lei et al. [24]	Enoxaparin 0.4 ml SC once daily	Within 24–48 h after liver resection	Until 24 h prior to discharge	N/A

^*^Hepatectomy cohort alone

### Risk of bias and quality assessment

Of the included studies, four [22, 24–26] received a NOS score of 7, suggesting high quality, and low-risk of bias. Two studies, each, had NOS scores of 6 [9, 21] and 5 [10, 23] suggestive of high-risk of bias.

### Demography and clinical characteristics

Baseline demographic and clinical characteristics of the study population have been detailed in Table 1. There were no differences in age, or gender, between the PTP + MTP and only MTP groups, except in the study by Yamashita et al. [22] wherein the patients receiving PTP were older (*p* = 0.01) and had higher BMI (*p* < 0.01). Pooled data from six studies [9, 10, 21, 22, 25, 26] revealed that 3625 patients underwent hepatectomy for malignancy, while 283 patients were operated on for benign disease. Hayashi et al. [23] and Ming-lei et al. [24] did not differentiate patients undergoing hepatectomy based on the indication for surgery. Three studies [22, 25, 26] involved patients with cirrhosis alone, with majority of these patients belonging to CTP class A. While patients receiving PTP + MTP were similar to those not receiving PTP with respect to pre-operative MELD scores and platelet counts in the studies by Yamashita et al. [22] and Wang et al. [25], the PTP + MTP arm had significantly lower mean MELD scores (*p* = 0.001) and higher platelet counts (*p* = 0.001) in the study by Vivarelli et al. [26].

### Definition of Extent of Hepatectomy

The extent of resection and definition of major hepatic resection varied between the included studies. Vivarelli et al. [26] defined minor resection as resection of ≤ 1 segment and major when ≥ 2 hepatic segments were resected, whereas Ejaz et al. [21] defined major resections as resection of ≥ 3 segments. Nathan et al. [10] and Hayashi et al. [23] defined major resections as resection of ≥ 4 segments, while Reddy et al. [9] included only patients who underwent resection of ≥ 4 liver segments in their study. The proportion of patients undergoing major hepatectomy, as per the definition used in the individual studies, has been shown in Table 1.

### Perioperative variables and outcomes

Three studies [22, 24, 26] found no difference between the two groups in terms of operative duration (Table 3). In contrast, while Ejaz et al. [21] noted increased operative duration in the PTP + MTP group (4.9 vs 4.2 h, *p* = 0.001), Hayashi et al. [21] reported a reduced operative duration (546 vs 622.5 min, *p* < 0.0001). Five studies [21–24, 26] found no difference between the two groups in terms of intra-operative PRBC transfusions. Although Reddy et al. [9] noted a higher intra-operative blood loss in the MTP group (*p* < 0.001), there was no difference in the need for PRBC transfusion. Vivarelli et al. [26] found that patients in the MTP group were more likely to have received intra-operative FFP transfusions (Table 3).

**Table 3:** Clinical outcomes in individual studies (IOBT-Intra-operative blood transfusion; LoS- Length of stay; MTP- Mechanical Thromboprophylaxis; PTP- Pharmacological Thromboprophylaxis; VTE- Venous thromboembolism)

Study	PTP+MTP versus only MTP					
	Operative duration (minutes)	Blood loss and transfusion requirements	VTE episodes* (*n*, %)	Hemorrhagic complications (%)	LoS, days {median (IQR) or mean ± SD}	Mortality (*n*, %)
Vivarelli et al. [26]	261 ± 106/275 ± 83 (*p*= 0.32)	124.7ml (0−1500)/154.1ml (0−1200)^ꝣ^ (*p*= 0.346)	1 (0.63%)/1 (1.38%) (*p*= 0.53)	5 (3.18%)/1 (1.39%) (*p*= 0.38)	**-**	**-**
Reddy et al. [9]	-	73 (26.6%)/30 (20.8%)^χ^ (*p*= 0.19)	6 (2.2%)/9 (6.3%) (*p*= 0.03)	-	7 ± 4/7 ± 6 (*p*= 0.04)	13 (4.7%)/11 (7.6%) (*p*= 0.22)
Nathan et al. [10]	-	-	28 (2.1%)/ 27 (3.3%)	22 (1.7%)/ 14 (1.64%) (*p*= 0.5)	**-**	**-**
Yamashita et al. [22]	343 ± 151/350 ± 146 (*p*= 0.74)	4 (8%)/31(13.5%)^χ^ (*p*= 0.2)	1 (1.9%)/24** (10.5%) (*p*= 0.04)	1(1.9%)/1 (0.43%) (*p*= 0.79)	17 ± 15/16 ± 16 (*p*= 0.67)	0 (0%)/0 (0%) (*p*= 0.99)
Ejaz et al. [21]	4.9 (3.6–6.4)/4.2 (3.3–5.0)^£^ (p<0.001)	102 (22.9%)/35 (24.3%)^χ^ (*p*= 0.8)	23 (5.1)/5 (3.3%) (*p*> 0.05)	-	5 (4–8)/5 (4–6) (*p*= 0.27)	10(2.2%)/3 (2.1%)^¥^ (*p*= 0.9)
Hayashi et al. [23]	546±185/622.5±218.8 (*p*<0.0001)	2 (0–4)/2 (0–4)^€^ (*p*= 0.21)	-	18 (25%)/6 (9.09%) (*p*< 0.05)	**-**	**-**
Wang et al. [25]	-	-	1 (0.85%)/16 (13.79%) (*p*< 0.05)	-	**-**	**-**
Ming-lei et al. [24]	224±78/229±96 (*p*= 0.68)	-	2 (2.1%)/5 (5.2%) (*p*= 0.44)	5(5.2%)/4(4.2%) (*p*= 1.0)	19 (15.0−25.0)/15.5 (12.0−24.8) (*p*= 0.015)	**-**

^*^VTE episodes- Includes both DVT, PE and PVT

^**^23 episodes of PVT and 1 episode of PE

^¥^90-day mortality

^ꝣ^Median blood loss in ml (range)

^€^Median number of IOBT (range)

^χ^Number of patients requiring IOBT (%)

^£^Operative duration (in hours)

Three studies [9, 22, 24] reported overall complications and found no difference between the two groups. While Nathan et al. [10] identified no difference, Ejaz et al. [21] found that major complications (> CD 3) [51] were more common in the PTP + MTP group (7.7 vs 2.8%; *p* = 0.04). Hayashi et al. [23] did not find any difference with respect to grade C PHLF [52] between the two groups (3.0% vs 8.3%). Among the three studies [9, 21, 22] which reported on post-operative mortality, there was no difference between the two groups. While the length of hospitalization was not different between the two in two studies [21, 22], Reddy et al. [9] and Ming-lei et al. [24] noted a significantly shorter stay in the PTP + MTP group (Table 3). No study provided data on readmission rates.

Yamashita et al. [22] and Hayashi et al. [23] employed a protocol-based routine CT scans on POD 5–7 and 14, respectively to detect VTE events. The rest of the studies [9, 10, 21, 24–26] performed imaging only upon clinical suspicion of VTE.

In the studies by Reddy et al. [9] (2.2% vs. 6.3%, *p* = 0.03) and Wang et al. [25] (0.85 vs 13.79%; *p* < 0.05), patients receiving PTP + MTP had lower rates of post-operative VTE (Table 3). Further elucidating the mechanisms underlying this observation, Wang et al. [25] reported down-regulation of Plasma P-selectin (CD62P), lysosomal granule glycoproteins (CD63) and D-dimer levels in the PTP + MTP group. In contrast, in the studies by Vivarelli et al. [26] and Nathan et al. [10], PTP (receipt or timing) was not associated with a difference in VTE events. Ejaz et al. [21] noted the incidence of VTE increased from < 5 to 14.3% when they compared the rates of its overall incidence to that following major hepatectomy. PTP was not associated with a difference in the incidence of VTE events. Of note, the incidence of VTE was 14.3% in patients with a peak post-operative INR ≥ 1.5, compared to 3.6% in patients with a peak INR ≤ 1.5 [21]. Yamashita et al. [22] found that PTP reduced the incidence of PVT (2% vs 10%; *p* = 0.04), though there was no difference in the rates of DVT (0% vs 0%; *p* = 0.99) or PE (0% vs 0.4%; *p* = 0.63). In the study by Hayashi et al. [23], which included patients undergoing pancreatoduodenectomy (*n* = 186) and hepatopancreatoduodenectomy (*n* = 25) in addition to hepatectomy (*n* = 138), post-operative VTE was significantly lower in the PTP + MTP group versus the MTP alone group (2.9 vs. 7.7%; RR 0.37, 95% CI 0.14–0.99; *p* < 0.05). Interestingly, the affected veins included those involved in central venous catheterization (*n* = 7) and those related to an operative procedure, such as the portal (*n* = 6) and hepatic veins/IVC (*n* = 3) in this study [23].

PTP did not confer an increased risk of hemorrhagic complications in three studies [10, 22, 26] (Table 3). In the study by Hayashi et al. [23], rates of post-operative hemorrhage were significantly higher with the use of PTP (25 vs. 9.09%; relative risk (RR) 2.75, *p* < 0.05), but only for minor hemorrhage (19.4 vs. 4.5%; RR 4.28, *p* < 0.05). The rates of major hemorrhage requiring blood transfusion and/or hemostasis with angiographic, or surgical, intervention, were not significantly different between the two groups (5.6 vs. 9.1%; RR 1.22, *p* > 0.05). Although no patient required interventions for hemorrhage, Ejaz et al. [21] reported a higher rate of post-operative PRBC transfusion (22.7 vs 5.0%; *p* < 0.001) in the PTP + MTP group. In contrast, Reddy et al. [9] reported higher post-operative transfusion requirements (46 (16.7%) vs 38 (26.4%); *p* = 0.02) in the MTP group, which might reflect an institutional practice of withholding PTP in patients who would have received transfusions.

### Meta-analysis

#### Post-operative VTE

**Fig. 2:**
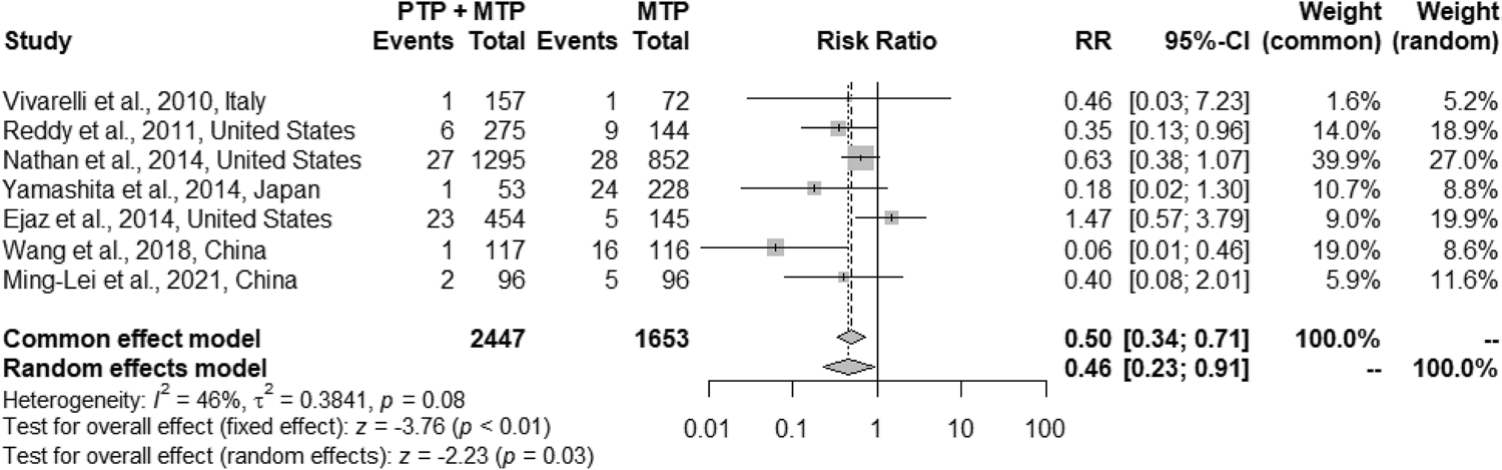
Forest plot comparing the effect of PTP + MTP versus only MTP on VTE events

Data on post-operative VTE as primary outcome was extracted from 7 studies [9, 10, 21, 22, 24–26]. Analysis of 4100 patients showed that PTP was associated with significantly lower rates of post-operative VTE (2.5% vs 5.3%; pooled RR 0.50, 95% CI: 0.34–0.71; *p* < 0.01), with moderate heterogeneity (*I*^2^ = 46%, *p* = 0.08) on the fixed-effects model (Fig. 2). In order to address heterogeneity, we performed an influential analysis using Baujat plot which depicts the contribution of each study to the overall *Q*-test statistic for heterogeneity on the horizontal axis versus the influence of each study on the vertical axis. The graphical method showed that while the study by Wang et al. [25] was the major source of overall heterogeneity, the findings of Ejaz et al. [21] had the maximal influence on the overall result (Supplementary Fig. 1). On excluding the study by Wang et al. [25], overall heterogeneity reduced to 20% (Supplementary Fig. 2a and b). Using TSA, the required information size was calculated at 4416 patients. The *Z*-curve crossed the conventional boundaries in favor of PTP before the information size was reached (Supplementary Fig. 3a), and the penalized *Z* value remained greater than 1.96, implying that the meta-analysis was conclusive with minimal risk of type 1 error (Supplementary Fig. 3b).

#### Post-operative hemorrhagic complications

**Fig. 3:**
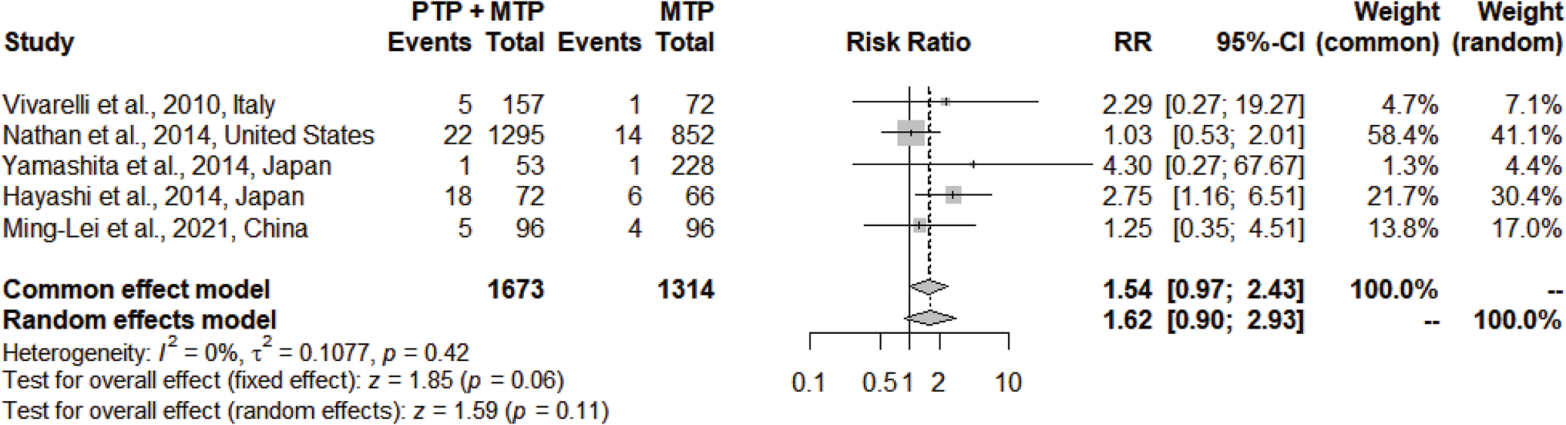
Forest plot comparing the effect of PTP + MTP versus only MTP on post-operative bleeding

The analysis of 2987 patients from 5 studies [10, 22–24, 26] showed no significant difference in the rates of post-operative bleeding with PTP (3.04% vs 1.9%; pooled RR 1.54, 95% CI: 0.97–2.43, *p* = 0.06) with no heterogeneity (*I*^2^ = 0%, *p* = 0.42) on the fixed-effects model (Fig. 3).

#### Post-operative length of stay

**Fig. 4:**
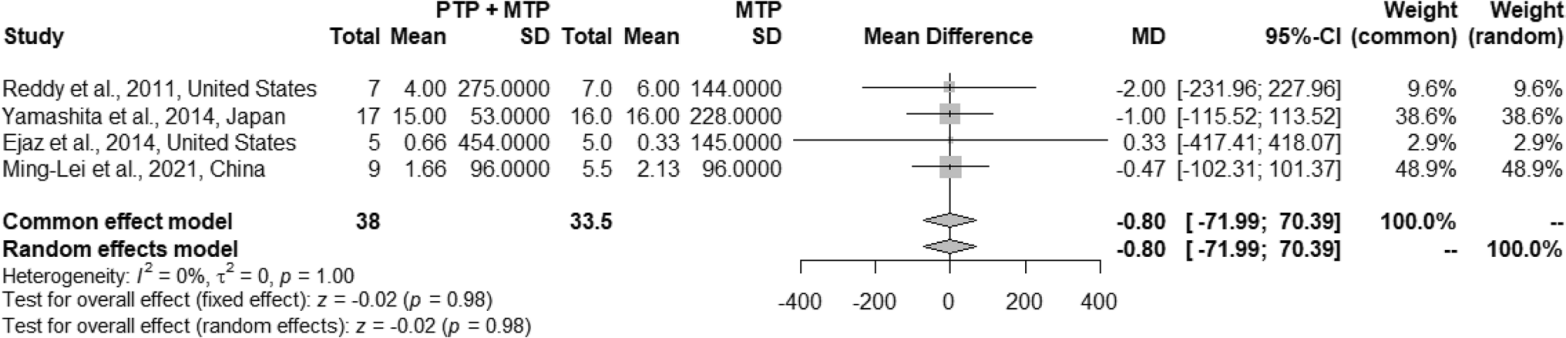
Forest plot comparing the effect of PTP + MTP versus only MTP on post-operative length of stay

An analysis of 1491 patients from 4 studies [9, 21, 22, 24] showed that there was no statistically significant difference in the rates of post-operative length of hospital stay with, or without PTP post-hepatectomy (mean 12.1 vs 10.8 days with pooled MD − 0.66, 95% CI: − 54.51 to − 53.2, *p* = 0.98), without any heterogeneity (*I*^2^ = 0%, *p* = 0.98) using the fixed-effects model (Fig. 4). Funnel plots (Supplementary Fig. 4) revealed no publication bias.

## Discussion

**Fig. 5:**
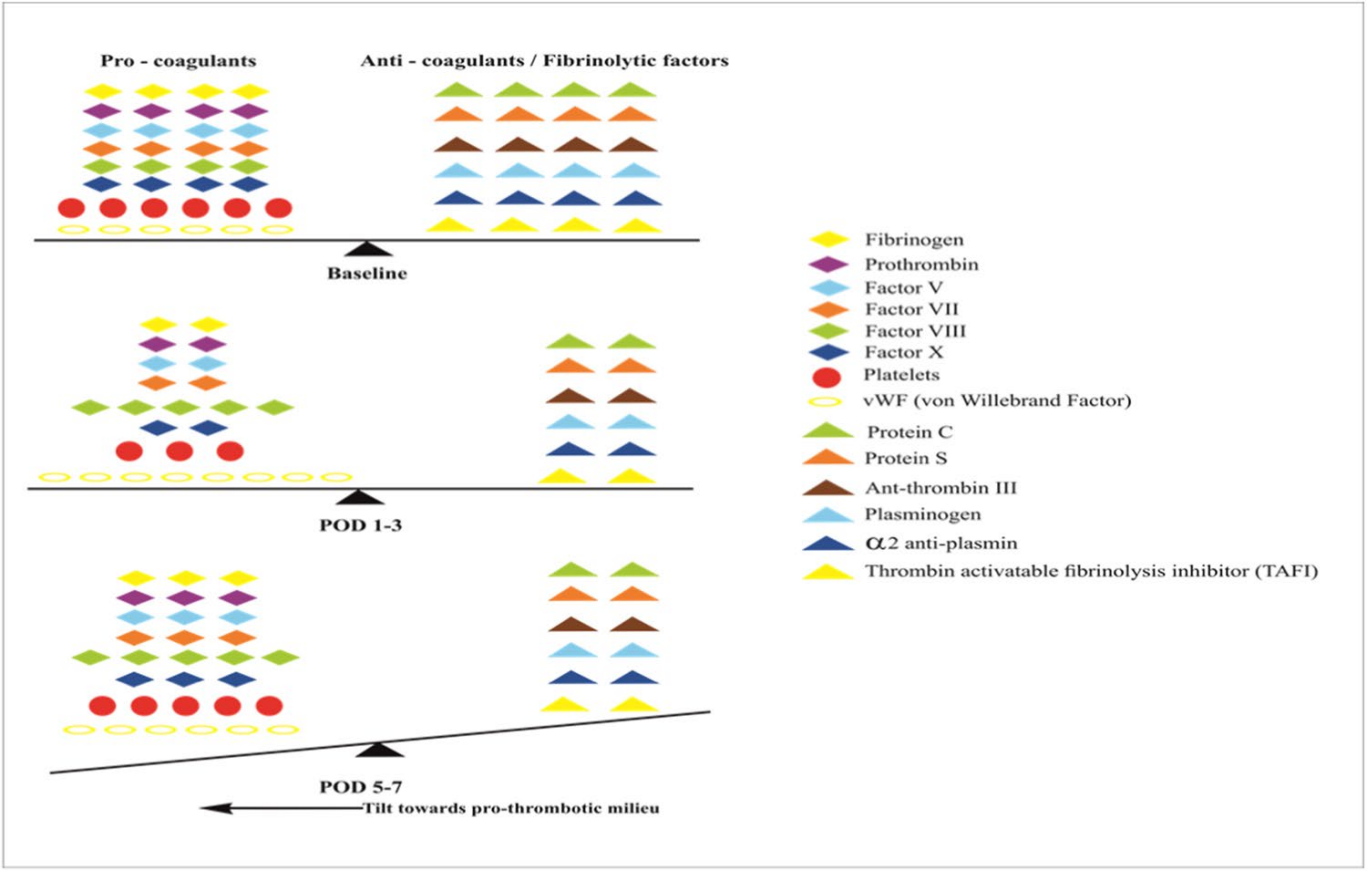
Coagulopathic perturbations following liver resection: Immediately following liver resection, there is a decrease in both pro- and anti-coagulant factors. However, faster recovery of the pro-coagulant factors, increased levels of vWF in the immediate post operative period, and increase in the factor VIII/protein C ratio renders the milieu pro-thrombotic [36, 37, 54–58]

Hepatectomy can precipitate a transient insufficiency in all liver functions, most notably, the synthetic function. This manifests as post-operative coagulopathy. The resultant coagulopathy is understood to be *dynamic*, on account of a continual flux in internal milieu from hypo-or hyper-coagulability, to normo-coagulability due to the complex interaction between pro- and anti-coagulant factors whose degree of reduction is varied and may be influenced by external factors such as the use of blood transfusions, and *functional* as it is an evanescent period of shifting balance in physiology, ad interim to a normal state of coagulation [53, 54]. Figure 5 depicts coagulopathic perturbations seen in the immediate post-operative period post-hepatectomy [36, 37, 54–58].

In the post-hepatectomy period, the risk of VTE outweighs the risk of bleeding proportionate to the magnitude of resection, especially in those who undergo extended resections, or who sustain post-operative complications [8, 59]. Of note, open hepatectomy is associated with higher VTE rates compared to minimally invasive hepatectomy (2.4% vs. 1.1%, *p* = 0.003), both for minor (1.9 vs. 1.0%, *p* = 0.028) and major liver resections (5.0 vs. 1.9%, *p* = 0.045) in a propensity matched analysis using ACS-NSQIP data [32]. This said, one should not forget that the quality of data on efficacy and safety of PTP is far from optimal, owing to the retrospective nature of the studies, heterogeneity in the indications, and patient population. In the current meta-analysis, we found that PTP + MTP was associated with significantly lower rates of post-hepatectomy VTE (2.5% vs 5.3%; pooled RR 0.50, 95% CI: 0.34–0.71; *p* = 0.03), with moderate heterogeneity (*I*^2^ = 46%, *p* = 0.08) and minimal type I error on TSA. However, this result should be interpreted with caution as the protection afforded by PTP was statistically insignificant in two individual studies [21, 26], while the study by Nathan et al. [10] had low power (41%) to detect a statistically significant difference at α = 0.05. A closer look at individual studies reveals that Wang et al. [25] reported significantly higher VTE rates of 13.79% in the MTP alone arm compared to other studies, and sensitivity analysis after exclusion of the study by Wang et al. [25, 59], reduced the heterogeneity to 20%. Nonetheless, two findings that strongly support the institution of PTP following hepatectomy are occurrence of PVT [22], and thrombosis in the veins involved in central venous catheterization or surgical procedure viz. portal vein and hepatic veins/ IVC [22, 23, 60], both of which cannot be prevented by MTP [22, 32]. This supports the important role of endothelial injury perpetrated by the invasive intervention in addition to the hypercoagulable state that ensues in the post-operative period [61]. The risk of PVT is increased with pre-existing cirrhosis, increased duration of Pringle maneuver, larger, especially right-sided resections, increased operative duration, and concomitant portal vein resection-reconstruction [62, 63]. We chose to include studies reporting PVT, as the intention of this systematic review was to examine the efficacy and safety of PTP post-hepatectomy irrespective of the pathogenetic mechanism of the VTE event. Its highly imperative to prevent post-operative PVT as it is a life-threatening condition and is reported to be associated with delayed liver regeneration with resultant delayed functional recovery [63].

There is a lack of clear understanding on the magnitude of risk of hemorrhage conferred by PTP following hepatic resections. While Minami et al. [35] noted that PTP did not increase the risk of hemorrhage after HPB surgeries, Fujikawa [64] found an elevated risk of bleeding in patients receiving PTP, with rates of overall, and major, bleeding complications ranging from 5.2 to 26.6% and 1.6 to 10.9%, respectively. In the current analysis, PTP did not confer an increased risk of hemorrhagic complications (3.04% vs 1.9%; pooled RR 1.54, *p* = 0.11) with no heterogeneity (*I*^2^ = 0%, *p* = 0.42). Again, bleeding events in the studies by Vivarelli et al. [26] and Yamashita et al. [22] could be controlled with conservative measures (usually cessation of PTP and blood product transfusion), without requiring an invasive (angiographic or surgical) intervention. Hayashi et al. [23] reported a significantly increased rate of minor post-operative hemorrhage in patients receiving PTP. They did not find an increased probability of major bleeding (being defined in the study based on the need for PRBC transfusions or angiographic or surgical intervention). It is highly important that future studies should strictly adhere to the ISGLS definition [65] for post-hepatectomy hemorrhage while reporting bleeding events, to enable comparison and pooling of the results from different studies.

The current study fails to arrive at a conclusion regarding the impact of use of PTP on post-operative mortality on account of small sample sizes in the included studies and low event rates. Similarly, from a morbidity perspective, there was no difference between the two groups in most of the included studies. VTE events have been shown to be associated with a significant increase in the length of hospitalization [8, 12], and any measure to reduce VTE events may intuitively be expected to bring about a reduction in the length of stay. However, the current study failed to discern a significant difference in length of stay between the PTP + MTP and MTP alone groups. In the absence of data on readmission rates within the included studies, we were unable to determine the impact of PTP on readmission rates.

There is clear lack of consensus [66, 67] regarding the optimal timing for the initiation of PTP (including whether it should be pre-, or post-operative), as well, the duration of PTP in patients undergoing hepatectomy. Generally, one of the following three regimens is followed: Post-operative dosing alone, peri-operative (pre-, as well as post-operative) dosing and extended dosing lasting for weeks after discharge [68]. Doughtie et al. [47] found that peri-operative PTP reduced the incidence of VTE (1.1% vs 6.1%, *p* = 0.05), but at increased risk of post-operative bleeding (10.9% vs 3.1%, *p* = 0.026) requiring intervention (reoperation, angiographic embolization, or percutaneous drainage for hematoma) in complex HPB surgeries. In contrast, Ainoa et al. [34] noted a significant reduction in VTE events (1.2% vs 9.7%, *p* < 0.0001), especially PE (1.2% vs. 9.3%, *p* < 0.0001), without any increase in hemorrhagic (*p* = 0.7186) or overall complications (*p* = 0.98) with pre-operative initiation of PTP.ERAS® society endorses initiation of LMWH or UFH 2–12 h before surgery, particularly in major hepatectomies [69].

Despite the knowledge that about one-third of VTE episodes occur post-discharge [33], routinely prescribing PTP in their discharge advice remains sub-optimal among HPB surgeons, though it seems to have increased from 14% in 2014 [41] to 39% by 2019 [70]. This inertia towards change despite evidence to the contrary [70] stems from the deeply entrenched fear of bleeding complications. Using a statistical model, Beal et al. [33] proposed that the Caucasian race, higher BMI, previous PCI, higher preoperative bilirubin, longer operative duration, perioperative transfusions, and re-operations conferred higher risk of post-discharge VTE and these patients were most likely to benefit from extended PTP. Further proof for safety of this approach comes from the study by Kim et al. [27], who reported no major bleeding events with extended PTP. Following extended PTP implementation, Lemke et al. [48] reported 81.4% perfect patient adherence with the regimen, further adding fuel to the thought that it is the physician reluctance which is the major deterrent to more widespread use of PTP.

We acknowledge the limitations of the study. There is significant heterogeneity in the study population not only in terms of the indication for surgery (benign versus malignant), but also in the PTP regimens (including the pooled analysis), the type and extent of liver resections, the presence of underlying chronic liver disease, and prior utilization of chemotherapy. It is believed that cirrhosis confers an increased risk of thrombotic events. However, among the included studies, majority of the cirrhotic patients belonged to CTP class A, and except in the study by Wang et al. [25], there were no documented coagulopathic disturbances in either group based on conventional preoperative testing. The retrospective nature of the data renders it highly probable that the decision to initiate PTP would have been impacted by intra-operative factors such as complexity of resection, adequacy of hemostasis, and/or hemoglobin levels. As only 2 studies implemented routine screening for VTE, the primary outcome consists predominantly of symptomatic VTE, which suffers from high risk of underreporting the actual number VTE episodes, though radiologic follow-up to identify asymptomatic events is rare in the post-operative VTE literature [71]. The studies fail to consistently furnish information on the severity of VTE (e.g., saddle pulmonary embolism vs. asymptomatic distal lower extremity DVT) and bleeding events. In the absence of the data from the included studies, we were unable to determine, through subgroup analysis, the impact of the type of PTP regimen utilized either in the preoperative setting or post-operative (including extended prophylaxis). Low event rates in the included studies might have rendered them underpowered to examine the true benefit of PTP to reduce VTE events [21]. The differences in the PTP regimens, extent, and approach for hepatectomy as well as heterogeneity in the pooled analysis may render it difficult to draw practice-altering conclusions from the available literature. However, the performance of trial sequential analysis to determine the precision and conclusiveness of the results in the presence of heterogeneity supports the efficacy of PTP in preventing VTE events without significantly increasing the risk of bleeding complications. A multi-institutional analysis with more granular and consistent data would shed better light on the topic and the question.

Nevertheless, the meta-analysis does not fail to support the role of peri-operative PTP in decreasing VTE events post-hepatectomy, despite its inherent limitations. In the light of knowledge that conventional testing does not substantiate the true magnitude of risk of thrombotic events, it is quite apparent that a “one size fits all” for VTE prophylaxis may not be the best approach. Beyond a need for greater utilization of visco-elastic point of care tests like TEG and ROTEM, we need to explore additional testing for better risk stratification. There is a pressing need for larger, well-designed studies examining the efficacy and safety of PTP, with special emphasis on timing of initiation and duration of prophylaxis. Future guidelines should take into consideration the racial differences and suggest a tailored approach as the risk–benefit ratio of post-operative VTE prophylaxis for Asians is roughly three times higher than that for Caucasians [72].

## Conclusions

Despite differences in the baseline patient characteristics, extent of hepatectomy, PTP regimens, and heterogeneity in the pooled analysis, the current study supports the use of PTP in post-hepatectomy patients (grade of recommendation: strong) as the combination of PTP + MTP is associated with a significantly lower incidence of VTE (level of evidence, moderate), without an increased risk of post-hepatectomy hemorrhage (level of evidence, low).

## Supplementary Information

Below is the link to the electronic supplementary material.

Supplementary file1 (DOCX 1869 KB)

Supplementary file2 (DOCX 43 KB)

Supplementary file3 (DOCX 63 KB)

Supplementary file4 (DOCX 390 KB)

Supplementary file5 (DOCX 13 KB)

Supplementary file6 (DOCX 17 KB)

## Abbreviations

ACS-NSQIP: American College of Surgeons National Surgical Quality Improvement Program
aPTT: Activated partial thromboplastin time
BMI: Body mass index
CD: Clavien-Dindo (grade of complications)
CT scan: Computed tomography scan
CTP: Child-Turcotte-Pugh Score
DVT: Deep vein thrombosis
ERAS®: Enhanced Recovery After Surgery
FFP: Fresh frozen plasma
HPB: Hepato-Pancreato-Biliary
INR: International normalized ratio
IS: Information size
ISGLS: International Study Group of Liver Surgery
IVC: Inferior vena cava
LMWH: Low molecular weight heparin
MELD: Model for end stage liver disease
MeSH: Medical education subject Headings
MTP: Mechanical thromboprophylaxis
PCI: Percutaneous coronary intervention
PE: Pulmonary embolism
PHLF: Post-hepatectomy liver failure
POD: Post-operative day
PRBC: Packed red blood cells
PRISMA: Preferred Reporting Items for Systematic Reviews and Meta-analyses
PT: Prothrombin time
PTP: Pharmacological thrombo-prophylaxis
PVT: Portal vein thrombosis
ROTEM: Rotational thromboelastometry
RR: Relative risk
TEG: Thromboelastography
TSA: Trial sequential analysis
UFH: Unfractionated heparin
VTE: Venous thromboembolism
