# Supplementary material for: Post-hepatectomy venous thromboembolism: a systematic review with meta-analysis exploring the role of pharmacological thromboprophylaxis
Source: Langenbecks Arch Surg. 2022 Jul 26;407(8):3221–33. doi: 10.1007/s00423-022-02610-9 (PMC9722838; doi:10.1007/s00423-022-02610-9)
Supplement: Supplementary file 1 — Supplementary file1 (DOCX 1869 KB) [file 423_2022_2610_MOESM1_ESM.docx]

**Supplementary Figure 1: Influential Analysis using Baujat plot (Overall heterogeneity (Q-statistic) versus the influence of individual studies)**

**
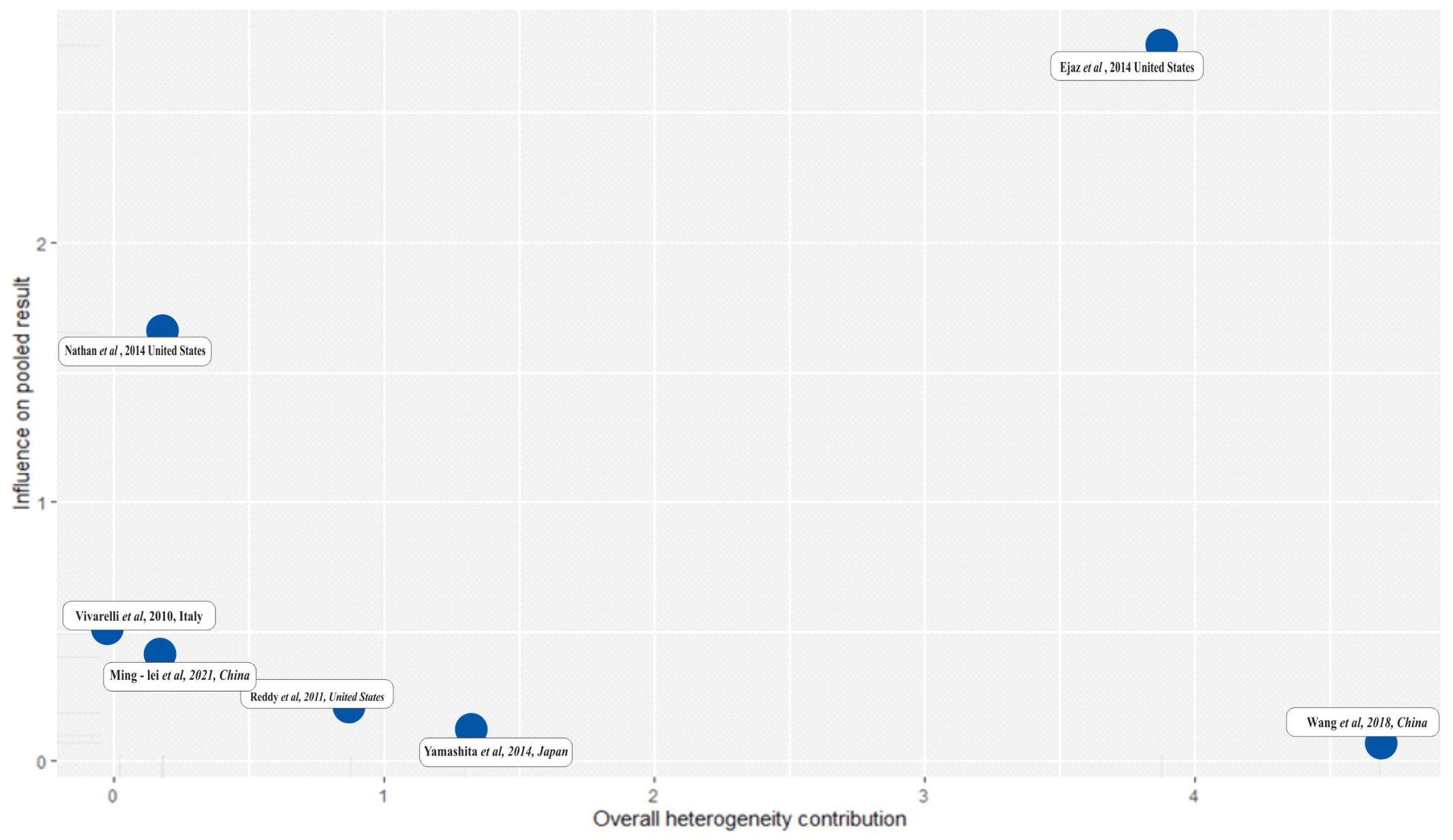
**
