# Supplementary material for: Post-hepatectomy venous thromboembolism: a systematic review with meta-analysis exploring the role of pharmacological thromboprophylaxis
Source: Langenbecks Arch Surg. 2022 Jul 26;407(8):3221–33. doi: 10.1007/s00423-022-02610-9 (PMC9722838; doi:10.1007/s00423-022-02610-9)
Supplement: Supplementary file 2 — Supplementary file2 (DOCX 43 KB) [file 423_2022_2610_MOESM2_ESM.docx]

**Supplementary Figure 2: Leave out analysis**

**Figure 2a: Leave out analysis sorted by effect size**


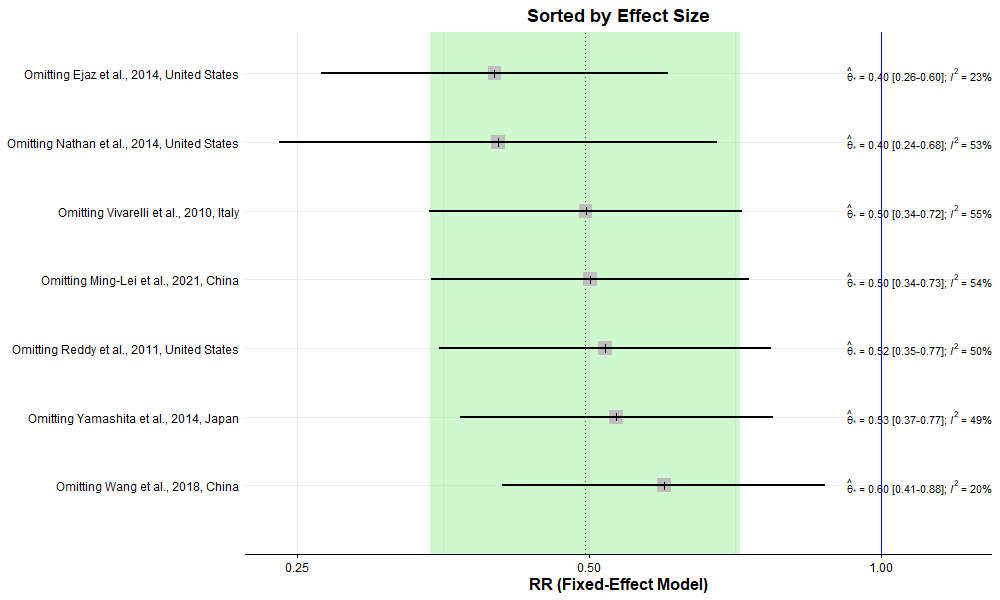


**Figure 2b: Leave out analysis sorted by heterogeneity (I^2^)**

**
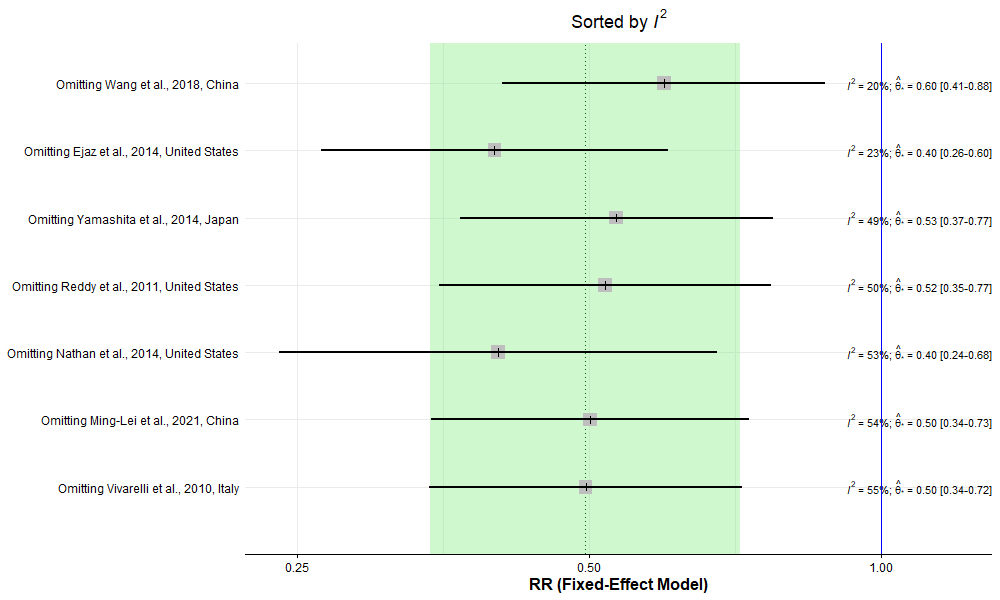
**
