# Supplementary material for: Post-hepatectomy venous thromboembolism: a systematic review with meta-analysis exploring the role of pharmacological thromboprophylaxis
Source: Langenbecks Arch Surg. 2022 Jul 26;407(8):3221–33. doi: 10.1007/s00423-022-02610-9 (PMC9722838; doi:10.1007/s00423-022-02610-9)

**Supplementary Figure 3a: Trial sequential analysis for post-operative VTE**

**
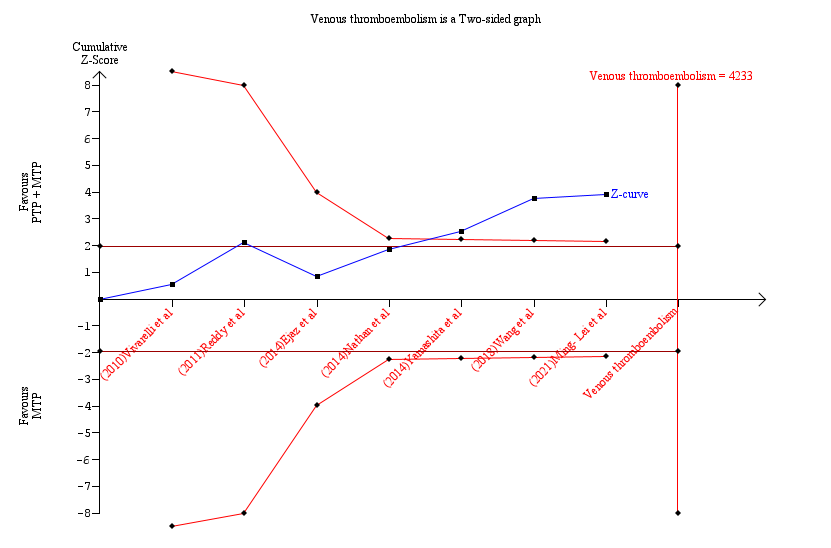
**

**Supplementary Figure 3b: Trial sequential analysis for post-operative VTE- Penalized Z curve**
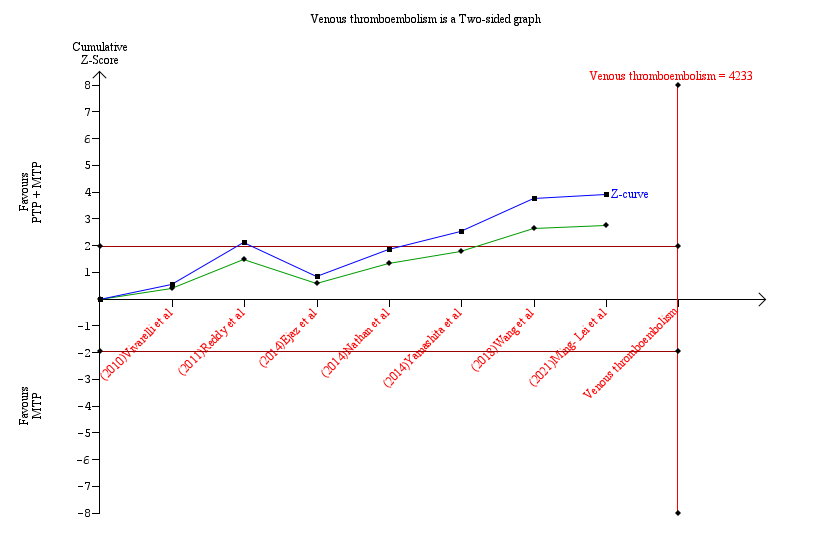

Supplement: Supplementary file 3 — Supplementary file3 (DOCX 63 KB) [file 423_2022_2610_MOESM3_ESM.docx]
