# Supplementary material for: Post-hepatectomy venous thromboembolism: a systematic review with meta-analysis exploring the role of pharmacological thromboprophylaxis
Source: Langenbecks Arch Surg. 2022 Jul 26;407(8):3221–33. doi: 10.1007/s00423-022-02610-9 (PMC9722838; doi:10.1007/s00423-022-02610-9)

**Supplementary Figure 4: Funnel Plots for publication bias (Abbreviation: VTE – venous thromboembolism)**


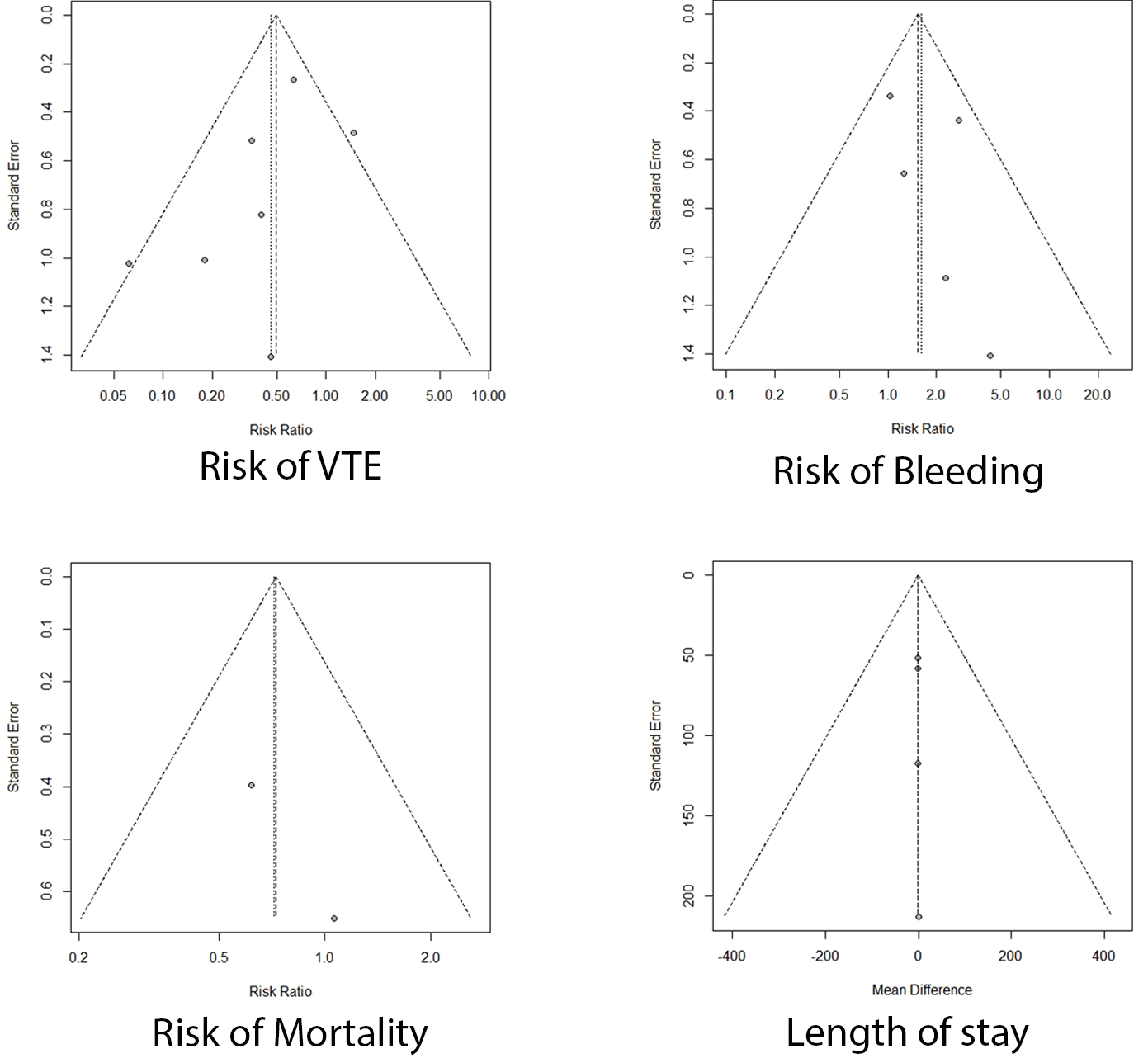

Supplement: Supplementary file 4 — Supplementary file4 (DOCX 390 KB) [file 423_2022_2610_MOESM4_ESM.docx]
