# Supplementary material for: Post-hepatectomy venous thromboembolism: a systematic review with meta-analysis exploring the role of pharmacological thromboprophylaxis
Source: Langenbecks Arch Surg. 2022 Jul 26;407(8):3221–33. doi: 10.1007/s00423-022-02610-9 (PMC9722838; doi:10.1007/s00423-022-02610-9)
Supplement: Supplementary file 5 — Supplementary file5 (DOCX 13 KB) [file 423_2022_2610_MOESM5_ESM.docx]

**Supplementary Table 1: Search strategy and combination of MeSH terms for PubMed/Medline**

| **Search** | **Search terms** | **Number of results** |
| --- | --- | --- |
| **#1** | *"Venous thromboembolism" OR “Deep vein thrombosis” OR “Pulmonary Embolism” OR “Thromboembolic episode”* | 87679 |
| **#2** | *“Hepatectomy" OR "Hepatic Resection" OR “Liver Resection”* | 5279 |
| **#3** | *“Thromboprophylaxis” OR “Pharmacological Thromboprophylaxis” OR "Chemothromboprophylaxis"* | 1027 |
| **#4** | *" Thromboprophylaxis” OR “Mechanical Thromboprophylaxis”* | 518 |
| **#5** | # 1 and # 2 and # 3 and # 4 | 7 |
