# Supplementary material for: Post-hepatectomy venous thromboembolism: a systematic review with meta-analysis exploring the role of pharmacological thromboprophylaxis
Source: Langenbecks Arch Surg. 2022 Jul 26;407(8):3221–33. doi: 10.1007/s00423-022-02610-9 (PMC9722838; doi:10.1007/s00423-022-02610-9)
Supplement: Supplementary file 6 — Supplementary file6 (DOCX 17 KB) [file 423_2022_2610_MOESM6_ESM.docx]

**Supplementary Table 2: Excluded studies and reasons for their exclusion (Abbreviations: VTE – venous thromboembolism; PTP- Pharmacological Thromboprophylaxis)**

| **Author [Year]** | **Reason for exclusion** |
| --- | --- |
| Kim et al. [2017] | No comparator group; the study analysed the safety of extended PTP till 14 or 28 days following surgery |
| Louis *et al*. [2014] | Did not evaluate the intervention of interest- administration of PTP |
| Chen *et al.* [2021] | Did not evaluate the intervention of interest- administration of PTP |
| Garg *et al.* [2021] | Did not evaluate the intervention of interest- administration of PTP |
| Newhook *et al.* [2015] | Did not evaluate the intervention of interest- administration of PTP |
| Mavros *et al.* [2021] | Did not evaluate the intervention of interest- administration of PTP |
| Hue *et al.* [2021 | Did not evaluate the intervention of interest- administration of PTP |
| Beal *et al.* [2018] | Did not evaluate the intervention of interest- administration of PTP |
| Ainoa *et al.* [2021] | No comparator arm, the study compared pre- versus post-op initiation of PTP |
| Minami *et al.* [2019] | No comparator arm |
| Barton *et al.* [2013] | Did not have intervention of interest, i.e. administration of PTP, or reported on outcomes, such as, VTE events or bleeding. |
| Kleiss *et al.* [2016] | Did not evaluate the intervention of interest- administration of PTP |
| Tzeng *et al.* [2012] | Did not evaluate the intervention of interest- administration of PTP |
| Singh *et al*. [2017] | Did not evaluate the intervention of interest- administration of PTP |
| Tzeng et al. [2013] | Did not evaluate the intervention of interest- administration of PTP |
| Raj *et al.* [2020] | Did not evaluate the intervention of interest- administration of PTP |
| Weiss *et al*. [2014] | No comparator arm, the study looked at practice patterns (regarding thromboprophylaxis) among American surgeons |
| Melloul *et al.* [2012] | Non-comparative study; Did not evaluate the intervention of interest- administration of PTP |
| Blasi *et al.* [2018] | Did not evaluate the intervention of interest- administration of PTP |
| Hoshikawa *et al.* [2018] | Abstract form, complete article not available |
| Schlick *et al.* [2021] | Non-comparative study; Did not evaluate the intervention of interest- administration of PTP |
| Marley *et al*. [2019] | No comparator arm, the study looked at adherence to extended TP |
| Doughtie *et al.* [2014] | Compared pre- versus post-operative TP |
| Lemke et al. [2016] | No comparator arm, the study looked at adherence to extended TP |
| Eguchi *et al.* [2020] | No comparator arm |
| Pitakteerabundit *et al.* [2022] | Awaiting peer-review, publication of the final manuscript. |
